# Lessons from Catalonia: advancing sexual and reproductive rights through feminist policymaking and governance

**DOI:** 10.1080/26410397.2025.2529072

**Published:** 2025-07-04

**Authors:** Tània Verge, Montserrat Pineda-Lorenzo

**Affiliations:** a MP in the Parliament of Catalonia, and former Minister of Equality and Feminisms of the Government of Catalonia (2021-2024), Professor of Politics and Gender, Universitat Pompeu Fabra (Barcelona), Barcelona, Spain.; b Social activist for sexual and reproductive rights, and former Secretary for Feminisms of the Government of Catalonia (2021-2024), Barcelona, Spain

**Keywords:** feminist and rights-based approach, abortion, contraception, menstruation and menopause, obstetric violence, transgender persons' healthcare, comprehensive sexuality education, pregnancy loss, feminist policymaking and governance, anti-gender movement

## Abstract

As sexual and reproductive rights (SRR) face mounting threats globally, Catalonia’s National Strategy for Sexual and Reproductive Rights showcases how democratic governments can expand SRR through feminist policymaking and governance. Building on the region’s robust public health infrastructure, systemic inequalities have been addressed and care standards improved with innovative policies. Grounded in intersectional feminism and a rights-based approach that prioritises bodily autonomy, universality, accessibility and participation, SRR have been embedded not only into health policies but also into education, workplaces and community frameworks. Key lessons from this experience highlight the importance of feminist expertise and leadership in driving impactful change. Framing SRR as human rights broadens their scope and facilitates collaboration across cabinet portfolios, while institutionalising policies within extant frameworks and services seeks to ensure their sustainability beyond political cycles. Furthermore, transformative SRR policies need not be cost-prohibitive; redesigning public services and training health professionals to deliver empathetic care can also yield significant results. Public engagement and participatory policymaking empower citizens and stakeholders, fostering accountability and a culture of advocacy. Catalonia’s bold and inclusive model, coupled with robust responses to the anti-gender movement, offers a blueprint for governments and advocacy organisations worldwide. [192]

## Résumé

Alors que les droits sexuels et reproductifs sont confrontés à des menaces croissantes dans le monde, la Stratégie nationale catalane pour les droits sexuels et reproductifs illustre comment des gouvernements démocratiques peuvent étendre ces droits grâce à la définition de politiques et la gouvernance féministes. Se fondant sur la solide infrastructure de santé publique de la région, des inégalités systémiques ont été corrigées et les normes de soins améliorées avec des politiques novatrices. Basés sur un féminisme intersectionnel et une approche fondée sur les droits qui privilégie l’autonomie corporelle, l’universalité, l’accessibilité et la participation, les droits sexuels et reproductifs ont été intégrés, non seulement dans les politiques de santé, mais aussi dans l’éducation, sur les lieux de travail et dans les cadres communautaires. Les principales leçons de cette expérience soulignent l’importance de l’expertise et du leadership féministes pour stimuler d’importants changements. Encadrer les droits sexuels et reproductifs comme des droits humains élargit leur portée et facilite la collaboration entre les ministères, alors que l’institutionnalisation des politiques au sein des cadres et services existants vise à garantir leur pérennité au-delà des cycles politiques. Par ailleurs, les politiques transformatrices en matière de droits sexuels et reproductifs ne doivent pas être d’un coût prohibitif ; la refonte des services publics et la formation des professionnels de santé à la prestation de soins empathiques sont aussi des façons d’obtenir des résultats significatifs. L’engagement public et la définition participative des politiques autonomisent les citoyens et les parties prenantes, favorisant la redevabilité et une culture de plaidoyer. Le modèle audacieux et inclusif de la Catalogne, associé à des réponses vigoureuses au mouvement anti-genre, offre un modèle aux gouvernements et aux organisations de défense des droits dans le monde entier.

## Resumen

A medida que los derechos sexuales y reproductivos (DSR) enfrentan crecientes amenazas a nivel mundial, la Estrategia Nacional de Derechos Sexuales y Reproductivos de Cataluña muestra cómo los gobiernos democráticos pueden ampliar los DSR mediante la formulación de políticas y gobernanza feministas. Aprovechando la robusta infraestructura de salud pública de la región, se han abordado las desigualdades sistémicas y se han mejorado los estándares de atención con políticas innovadoras. Basados en el feminismo interseccional y en un enfoque de derechos que prioriza la autonomía corporal, la universalidad, la accesibilidad y la participación, los DSR han sido incorporados no solo en las políticas de salud, sino también en la educación, en lugares de trabajo y en marcos comunitarios. Las lecciones clave de esta experiencia destacan la importancia de la experticia y el liderazgo feministas para impulsar cambios impactantes. Al incluir DSR en el marco de los derechos humanos, se amplía su alcance y se facilita la colaboración entre las carteras de gabinetes, mientras que la institucionalización de políticas dentro de los marcos y servicios existentes busca garantizar su sostenibilidad más allá de los ciclos políticos. Asimismo, las políticas transformadoras sobre DSR no tienen que ser costo-prohibitivas; el rediseño de servicios públicos y la capacitación de profesionales de la salud en la prestación de servicios empáticos también pueden producir resultados significativos. El compromiso público y la formulación de políticas participativa empoderan a la ciudadanía y a las partes interesadas, fomentando la rendición de cuentas y una cultura de incidencia política. El modelo audaz e inclusivo de Cataluña, junto con respuestas robustas al movimiento antigénero, ofrece un plan para los gobiernos y las organizaciones de incidencia política a nivel mundial.

## Introduction

As sexual and reproductive rights (SRR) face increasing challenges and setbacks worldwide,^1,2^ Catalonia’s *National Strategy for Sexual and Reproductive Rights (NSSRR)* provides a beacon of hope. Building on an intersectional feminist and rights-based approach and founded on the principle of bodily autonomy, the NSSRR prioritises universality, accessibility and participation and addresses SRR throughout the life course, extending well beyond health by embedding SRR into education, workplaces and community activities.

This region of eight million inhabitants already had a robust sexual and reproductive public health infrastructure and the most advanced policies within Spain. In this multi-level system, regions can expand the provisions of the countrywide framework laws with their own legislation on competences shared with the national level of government. They are responsible for delivering basic public services such as health or education, often leading to significant differences across regions.^3^ Making use of its self-government capacities, the Government of Catalonia designed a comprehensive set of transformative SRR policies during the period 2021–2024 to address systemic inequalities, improve care standards and counter attacks from anti-gender groups.*

The leading actor in the NSSRR was the newly established Ministry of Equality and Feminisms. Its creation was an electoral pledge of the left-wing pro-independence party Republican Left of Catalonia, reflecting the mobilisation strength of the women’s movement in previous years. Many of its officeholders, including ourselves, were feminist social activists and academics. Drawing on our own experience in the said Ministry, this commentary outlines the NSSRR’s innovative measures, institutionalisation efforts and broader implications for the global SRR agenda and policymaking in this field. The Catalan case showcases how international norms^†^ can be translated into specific policies and services, demonstrating that feminist political action works.

## Holistic approach to SRR

The NSSRR included far-reaching policies that addressed both structural barriers and emerging challenges. Against a backdrop of declining abortion care infrastructure in several countries, abortion services (both medical and surgical) were expanded across the region to guarantee equitable access. As of 2024, about 40% of all abortion centres in Spain were located in Catalonia, which is one of the few regions with a registry of conscientious objection for healthcare professionals. The 2022 updated *Protocol for abortion care* extended medical abortion from 9 to 14 weeks of gestation, following WHO guidelines, and established psychological counselling, post-abortion comprehensive support, free long-term contraception and screenings for gender-based violence. Additionally, free copper IUDs and subdermal implants were introduced for women and transgender persons with gestational capacity under 30 years old and, regardless of age, for those with socioeconomic deprivation and survivors of gender-based violence. The 2023 *Action plan to improve access to long-term contraception* aims for universal, cost-free access to these methods by 2026.

In parallel, the *Menstrual and climacteric equity plan (2023–2025)* constitutes a global first in tackling period poverty, stigma, lack of information on our own bodies, and the environmental impact of single-use products. Between March 2024 and March 2025, about 510,000 free reusable menstrual products – a menstrual cup, a pair of panties or two cloth pads – had already been distributed to girls, women and other menstruating persons aged 10–60 years old through over 3000 local pharmacies, accompanied by counselling. Pharmacists received specific training by the Ministry of Equality and Feminism. To further promote menstrual health, education programmes were introduced in high schools. Similar programmes are run in women’s shelters, prisons and juvenile centres, where kits with reusable products are handed out. Additionally, since 2022, civil servants in Catalonia have benefited from an eight-hour recoverable monthly flexibility scheme to address health and well-being challenges associated with menstruation or the climacteric,^‡^ normalising workplace conversations and combating ageism.

Moreover, the global first *Plan to address obstetric violence and violations of SRR (2023–2028)* tackles inequities in maternal healthcare and, more generally, in sexual and reproductive health practices, as mandated by the modification of the Catalan Act on women’s right to eradicate gender-based violence, passed in 2020 – with such violations of rights being considered institutional violence when they occur in public services.^§^ This initiative has created hospital committees to monitor practices, reduce unnecessary caesarean sections and promote informed and autonomous decision-making. Funding for assisted reproductive technologies was increased to reduce waiting times. Meanwhile, a comprehensive training programme for healthcare professionals strengthens empathetic care, knowledge of LGBTI healthcare, early diagnosis of endometriosis, and perinatal mental health support. Healthcare facilities are also being adapted for people with disabilities or neurodivergence. In response to pregnancy loss, a three-day leave for losses occurring between week six and day 179 was introduced for civil servants – after this day, the Spanish Social Security accords maternity leave. The leave applies to both the pregnant person and their partner or a close relative.

To equip young people with knowledge about their sexual and reproductive health and rights and to promote respect and inclusivity, comprehensive sexuality education became mandatory in pre-school, primary and secondary education curricula in 2022. In 2023, a coordinator for gender-inclusive education and well-being was introduced in education centres. Counselling services are also provided in high schools through the School and Health Program, connecting students with Sexual and Reproductive Healthcare Units in primary healthcare centres.

Regarding efforts to improve transgender healthcare, non-pathologising units offering psychological support, hormonal treatments, surgeries, and counselling for families and support networks increased from four in 2021 to eight by 2024. Integrated into the public health system’s primary care, these units reduce barriers and stigma. Furthermore, to address discrimination against people living with HIV, the 2022 *Social Pact to Eradicate Discrimination* combats serophobia and ensures equitable access to public and private services, including life insurance and employment opportunities.

Finally, public engagement and research were central to the NSSRR. In 2023, the Catalan public broadcasting service devoted its health telethon to sexual and reproductive health, raising €8.2 million for 26 research projects. This edition broke participation records, with about one million people engaging in over 3,200 awareness events, including those hosted by 97% of secondary education centres. This reflects society’s eagerness to break stigmas on sexual and reproductive health and painful silences around long-neglected pathologies.

## Institutionalising feminist governance

With the Ministry of Equality and Feminisms standing on equal footing with the other cabinet ministries, the NSSRR was included as a core policy of the Government Plan, that is, the policy roadmap for the term passed in September 2021. This facilitated the creation of an all-government strategy, probably one of the few in the world, led by the said Ministry, which set up an interdepartmental committee – with the Ministry of Health as deputy chair – to coordinate all measures.

Indeed, a key concern was institutionalising SRR policies to endure beyond political cycles. This led to embedding the NSSRR into existing structures, such as integrating the Ministry of Health’s *Plan for affective, sexual, and reproductive health* into the NSSRR and securing the Ministry of Equality and Feminisms a seat on the committee monitoring *Catalonia’s Health Plan (2021-2025)*. Intertwining policies and structures ensured coherence, while multi-annual interdepartmental plans (menstrual equity, obstetric violence, etc.) with timelines, evaluation indicators and budgetary commitments will help hold future governments accountable.

Another key factor for the institutionalisation of the NSSRR has been the creation of alliances, multi-stakeholder partnerships and consultation processes with civil society organisations, scientific associations, health professionals, trade unions, academics and other relevant actors. This bottom–up approach ensures that policies address both immediate needs and evolving demands and that they reach all publics, including socioeconomically deprived population groups, such as undocumented migrants. It also reinforces their legitimacy and sustainability over time, as collective decision-making instils a sense of ownership.

For instance, the *Menstrual and Climacteric Action Plan* was designed in collaboration with feminist organisations specialising in the topic; its implementation involves women’s groups; while pharmacists have been highly engaged in outreach activities via social media and community workshops. The bid for the sexual and reproductive health telethon was prepared with professional, scientific and women’s associations devoted to increasing medical attention to neglected pathologies such as endometriosis. The Ministry’s two participatory councils – the Women’s National Council of Catalonia and the LGBTI National Council – contributed to the design of the new sexuality education curricula alongside civil society organisations experienced in school workshops. And feminist organisations joined a study mission with multilateral agencies and transnational reproductive justice organisations to evaluate the NSSRR, while transgender associations have long been engaged in the design and oversight of the non-pathologising healthcare units.

Similarly, ongoing training for health professionals and integrating policies into existing services aimed to support their institutionalisation. For example, the universal distribution of reusable menstrual products is facilitated through the Catalan public health app, which provides a QR code to collect the free products at pharmacies. This app is widely used for scheduling medical visits and accessing test results.

Likewise, the demand creation of rights instilled by the NSSRR is of paramount importance for the sustainability of policies over time. Once new rights are introduced, setbacks can yield political costs. Indeed, framing our policies in terms of rights has stimulated the sense of ownership of SRR rights by the Catalan population, empowering citizens to demand more policies – for instance, the launching of the free distribution of reusable menstrual products stirred women citizens’ demand for more counselling on the climacteric.

## Countering resistance and expanding public support

Institutional responses from democratic institutions to anti-gender groups are still few and far between.^5^ This challenge was addressed robustly by the Government of Catalonia. It intervened against a transphobic campaign run by the ultra-conservative group Hazte Oír, which is part of the Europe-wide anti-gender platform CitizenGO. The group was fined €20,000 and its bus, vinyled with discriminatory messages, was halted. Anti-gender groups also mobilised conservative families in educational centres to demand a non-existing right to opt out of sexuality education. In response, the Ministry of Education’s Inspection Service centralised replies, making it clear that non-attendance at curricular activities would be considered absenteeism. Additionally, the ultrareligious Spanish-wide association Christian Lawyers filed a lawsuit against the Government of Catalonia and requested a preliminary injunction to suspend the gender-inclusive educational programme. The court denied the injunction, but the final judgement is still pending. The Ministry of the Interior, on its part, established buffer zones around abortion clinics to protect both patients and healthcare providers from the harassment staged by the anti-rights international campaign “40 Days for Life”. No other government within Spain adopted such diligent responses.

The Ministry of Equality and Feminisms also designed preventive responses to counter the portrayal by the extreme-right party Vox – which at the time had 11 out of 135 seats in the Parliament of Catalonia – of the NSSRR policies as unnecessary, a waste of money, and “woke”. On the one hand, we employed strategic framing. For example, the right to menstrual equity was linked to issues of gender justice, social justice and climate justice, thereby broadening its appeal and legitimacy across different audiences. On the other hand, we grounded the policies in empirical evidence to build support, drawing on existing research, reports from international organisations and civil society associations, and public opinion surveys. The latter were particularly useful in highlighting gaps in societal knowledge on topics such as menstruation – including the prevalence of period poverty – and sexuality education.

Furthermore, the *Action plan to support human rights defenders* (2022), including SRR defenders, aimed to protect their vital work from retaliation by anti-gender groups. Besides collecting data on the anti-gender attacks to inform protective measures, training on self-protection tools and evidence collection to file criminal charges has been provided. Moreover, funding for human rights organisations’ projects was increased, covering awareness campaigns, research, strategic litigation, shadow reports for international organisations, and services for victims of attacks. Another key aspect of the plan is free legal advice to assess the reparation options.

## Lessons from the field

Catalonia’s experience offers relevant insights to policymakers and activists alike for advancing SRR. First, feminist expertise and leadership drive impactful change. Second, framing SRR as human rights broadens their scope and engages all cabinet portfolios. Third, embedding policies in institutional frameworks ensures their sustainability. Fourth, transformative SRR policies need not be costly; redesigning services and training professionals might well foster empathetic care. Fifth, public engagement is crucial, with participatory policymaking and the promotion of rights ownership empowering stakeholders and citizens while fostering accountability. Sixth, institutional responses to anti-gender movements and protection for SRR defenders reinforce democratic resilience.

That said, whereas several transformative policies were introduced in just three years, some challenges also arose. To start with, coordinating with other ministries required navigating their agendas and managing timing constraints, often amid crises such as sector-wide strikes. Similarly, significant effort was put into persuading some officeholders and top-ranking bureaucrats of the necessity of changing existing approaches or introducing – and funding – new policies. Launching ambitious policies while simultaneously building from scratch the young ministry’s structure was also very demanding, given the human and financial constraints that new portfolios face. However, this was offset by the drive, commitment and long hours of both the Ministry of Equality and Feminisms’ officeholders and personnel as well as feminist allies in other portfolios. They all deserve our deepest appreciation and recognition.

To conclude, regional governments can spearhead global SRR political agendas. The Catalan case exemplifies how feminist policymaking and governance can fulfil and expand these fundamental rights. Feminist governance is not only possible, even in the face of mounting opposition, but also indispensable to overcoming systemic barriers and discrimination. By ingraining an intersectional feminist perspective and human rights principles in all policies, Catalonia offers a blueprint for resistance and progress that can inspire both national and regional governments as well as advocacy organisations worldwide.

## Footnotes

* See: https://igualtat.gencat.cat/ca/ambits-dactuacio/drets-sexuals-reproductius/estrategia-nacional-de-drets-sexuals-i-reproductius/

† Universal Declaration of Human Rights, Convention on the Elimination of All Forms of Discrimination against Women (including CEDAW committee’s general recommendations), Beijing Platform for Action, Cairo Declaration on Population and Development and ICPD recommendations, Word Health Organization’s (WHO) statements and action plans, and the European Parliament’s and the Parliamentary Assembly of the Council of Europe’s (PACE) resolutions on sexual and reproductive health and rights.

‡
Measures for private sector workers can only be introduced through Spanish-wide legislation.

§
Hitherto, it is the only legal measure on obstetric violence enacted within the European Union. ^4^
